# Supplementary material for: A UMLS-based spell checker for natural language processing in vaccine safety
Source: BMC Med Inform Decis Mak. 2007 Feb 12;7:3. doi: 10.1186/1472-6947-7-3 (PMC1805499; doi:10.1186/1472-6947-7-3)
Supplement: Additional file 13 — RAP application directory. Sets up directory for RAP (RDF application for PHP) source code [file 1472-6947-7-3-S13.gz › rap/doc/phpdoc/default/OWL_RES.html]

xml version="1.0" encoding="iso-8859-1"?


Docs For Class OWL\_RES


## Class OWL\_RES

Description

Description |
Methods (details)

Located in /vocabulary/OWL\_RES.php (line 26)

Method Summary

Description |
Methods (details)

void
ALL\_DIFFERENT
()

void
ALL\_VALUES\_FROM
()

void
ANNOTATION\_PROPERTY
()

void
BACKWARD\_COMPATIBLE\_WITH
()

void
CARDINALITY
()

void
COMPLEMENT\_OF
()

void
DATATYPE
()

void
DATATYPE\_PROPERTY
()

void
DATATYPE\_RESTRICTION
()

void
DATA\_RANGE
()

void
DEPRECATED\_CLASS
()

void
DEPRECATED\_PROPERTY
()

void
DIFFERENT\_FROM
()

void
DISJOINT\_WITH
()

void
DISTINCT\_MEMBERS
()

void
EQUIVALENT\_CLASS
()

void
EQUIVALENT\_PROPERTY
()

void
FUNCTIONAL\_PROPERTY
()

void
HAS\_VALUE
()

void
IMPORTS
()

void
INCOMPATIBLE\_WITH
()

void
INTERSECTION\_OF
()

void
INVERSE\_FUNCTIONAL\_PROPERTY
()

void
INVERSE\_OF
()

void
MAX\_CARDINALITY
()

void
MIN\_CARDINALITY
()

void
NOTHING
()

void
OBJECT\_CLASS
()

void
OBJECT\_PROPERTY
()

void
OBJECT\_RESTRICTION
()

void
ONE\_OF
()

void
ONTOLOGY
()

void
ON\_PROPERTY
()

void
OWL\_CLASS
()

void
PRIOR\_VERSION
()

void
PROPERTY
()

void
RESTRICTION
()

void
SAME\_AS
()

void
SAME\_CLASS\_AS
()

void
SAME\_INDIVIDUAL\_AS
()

void
SAME\_PROPERTY\_AS
()

void
SOME\_VALUES\_FROM
()

void
SYMMETRIC\_PROPERTY
()

void
THING
()

void
TRANSITIVE\_PROPERTY
()

void
UNION\_OF
()

void
VERSION\_INFO
()

Methods

Description |
Methods (details)

ALL\_DIFFERENT (line 35)

void

ALL\_DIFFERENT
()

ALL\_VALUES\_FROM (line 41)

void

ALL\_VALUES\_FROM
()

ANNOTATION\_PROPERTY (line 29)

void

ANNOTATION\_PROPERTY
()

BACKWARD\_COMPATIBLE\_WITH (line 47)

void

BACKWARD\_COMPATIBLE\_WITH
()

CARDINALITY (line 53)

void

CARDINALITY
()

COMPLEMENT\_OF (line 65)

void

COMPLEMENT\_OF
()

DATATYPE (line 71)

void

DATATYPE
()

DATATYPE\_PROPERTY (line 77)

void

DATATYPE\_PROPERTY
()

DATATYPE\_RESTRICTION (line 89)

void

DATATYPE\_RESTRICTION
()

DATA\_RANGE (line 83)

void

DATA\_RANGE
()

DEPRECATED\_CLASS (line 95)

void

DEPRECATED\_CLASS
()

DEPRECATED\_PROPERTY (line 101)

void

DEPRECATED\_PROPERTY
()

DIFFERENT\_FROM (line 113)

void

DIFFERENT\_FROM
()

DISJOINT\_WITH (line 119)

void

DISJOINT\_WITH
()

DISTINCT\_MEMBERS (line 107)

void

DISTINCT\_MEMBERS
()

EQUIVALENT\_CLASS (line 125)

void

EQUIVALENT\_CLASS
()

EQUIVALENT\_PROPERTY (line 131)

void

EQUIVALENT\_PROPERTY
()

FUNCTIONAL\_PROPERTY (line 137)

void

FUNCTIONAL\_PROPERTY
()

HAS\_VALUE (line 143)

void

HAS\_VALUE
()

IMPORTS (line 155)

void

IMPORTS
()

INCOMPATIBLE\_WITH (line 149)

void

INCOMPATIBLE\_WITH
()

INTERSECTION\_OF (line 161)

void

INTERSECTION\_OF
()

INVERSE\_FUNCTIONAL\_PROPERTY (line 167)

void

INVERSE\_FUNCTIONAL\_PROPERTY
()

INVERSE\_OF (line 173)

void

INVERSE\_OF
()

MAX\_CARDINALITY (line 179)

void

MAX\_CARDINALITY
()

MIN\_CARDINALITY (line 185)

void

MIN\_CARDINALITY
()

NOTHING (line 191)

void

NOTHING
()

OBJECT\_CLASS (line 197)

void

OBJECT\_CLASS
()

OBJECT\_PROPERTY (line 203)

void

OBJECT\_PROPERTY
()

OBJECT\_RESTRICTION (line 209)

void

OBJECT\_RESTRICTION
()

ONE\_OF (line 215)

void

ONE\_OF
()

ONTOLOGY (line 227)

void

ONTOLOGY
()

ON\_PROPERTY (line 221)

void

ON\_PROPERTY
()

OWL\_CLASS (line 59)

void

OWL\_CLASS
()

PRIOR\_VERSION (line 233)

void

PRIOR\_VERSION
()

PROPERTY (line 239)

void

PROPERTY
()

RESTRICTION (line 245)

void

RESTRICTION
()

SAME\_AS (line 251)

void

SAME\_AS
()

SAME\_CLASS\_AS (line 257)

void

SAME\_CLASS\_AS
()

SAME\_INDIVIDUAL\_AS (line 263)

void

SAME\_INDIVIDUAL\_AS
()

SAME\_PROPERTY\_AS (line 269)

void

SAME\_PROPERTY\_AS
()

SOME\_VALUES\_FROM (line 275)

void

SOME\_VALUES\_FROM
()

SYMMETRIC\_PROPERTY (line 281)

void

SYMMETRIC\_PROPERTY
()

THING (line 287)

void

THING
()

TRANSITIVE\_PROPERTY (line 293)

void

TRANSITIVE\_PROPERTY
()

UNION\_OF (line 299)

void

UNION\_OF
()

VERSION\_INFO (line 305)

void

VERSION\_INFO
()

Documentation generated on Fri, 17 Dec 2004 16:16:48 +0100 by phpDocumentor 1.3.0RC3
